# Case Report: A family of fluctuating cystoid macular edema caused by *MYO7A* gene mutations

**DOI:** 10.3389/fmed.2025.1582930

**Published:** 2025-08-07

**Authors:** Cong Duan, Yuan Zong, Qian Chen, Wei Liu, Qing Chang, Cheng Xiong, Zhu-Lin Hu, Ge-Zhi Xu, Feng-Juan Gao

**Affiliations:** ^1^Yunnan Eye Institute & Key Laboratory of Yunnan Province, Yunnan Eye Disease Clinical Medical Center, Affiliated Hospital of Yunnan University, Yunnan University, Yunnan, China; ^2^Eye Institute and Department of Ophthalmology, Eye & ENT Hospital, Fudan University, Shanghai, China; ^3^Shanghai Key Laboratory of Visual Impairment and Restoration, Shanghai, China; ^4^NHC Key Laboratory of Myopia and Related Eye Diseases; Key Laboratory of Myopia and Related Eye Diseases, Chinese Academy of Medical Sciences, Shanghai, China

**Keywords:** cystoid macular edema, *MYO7A* gene, phenotype, genetic heterogeneity, optical coherence tomography

## Abstract

Cystoid macular edema (CME) is a common complication in various retinal disorders, often leading to significant central vision impairment. However, the underlying genetic causes and detailed clinical features in patients with fluctuating CME remain unclear. This retrospective, observational case series analyzed two patients from a single family with fluctuating CME, focusing on both clinical and genetic aspects. Data were collected and analyzed from September 2022 to January 2023 at a single center. Comprehensive ocular examinations, including best-corrected visual acuity tests, color fundus photography, fundus fluorescein angiography (FFA), optical coherence tomography (OCT), visual field tests, flash electroretinography, multifocal electroretinography, and electrooculography, were performed. Genetic analysis was conducted using whole exome sequencing, with confirmation through Sanger sequencing and co-segregation analysis. The results identified two compound heterozygous variants in the *MYO7A* gene: c.562C>G p.Q188E and c.5929C>T p.R1977W in both patients. Fundus fluorescein angiography revealed cystoid hyperfluorescence in a petaloid pattern in the foveal area and a honeycomb pattern parafoveally. OCT showed that macular cystoid changes were primarily located in the outer nuclear layer (ONL), and full-field electroretinography indicated rod-cone dysfunction. Over a 108-day follow-up period, CME in both patients exhibited fluctuating changes without any treatment. This case series suggests that the identified MYO7A variants are likely associated with fluctuating CME, expanding the phenotypic spectrum of MYO7A and providing new insights into the mechanisms underlying CME. Identifying these MYO7A variants bridges genetic research with clinical diagnostics, potentially offering more precise and personalized treatment strategies for retinal disorders.

## Introduction

Macular edema is a major cause of visual impairment in the course of several retinal diseases, such as diabetic retinopathy, retina vein occlusions, uveitis, and inherited retinal diseases (1). Cystoid macular edema (CME) can be defined as an abnormal accumulation of fluid in the neural retina layers and presents as cystoid spaces on optical coherence tomography (OCT) B-scans (2). Mechanisms associated with CME include breakdown of the inner and outer blood-retinal barrier, dysfunction of Müller or retinal pigment epithelium cells (3), and vitreous traction (4). Although the pathogenesis of CME is not clearly understood, it is a well-described feature of several inherited retinal diseases, such as retinitis pigmentosa (RP) [CME is observed in 10%−50% of RP patients (5)], juvenile X-linked retinoschisis, gyrate atrophy, and chloridemia (6).

*MYO7A* (OMIM: 276903) is located at 11q13.5 and has 49 exons. It encodes an unconventional myosin of 2,215 amino acids, myosin VIIA (7), which is a member of the large myosin family. Myosin VIIA plays an important role in retinal photoreceptor cells, the pigment epithelium and inner ear; therefore, mutations in *MYO7A* result in a spectrum of phenotypes ranging from Usher syndrome type 1B (8) to recessive non-syndromic hearing loss (DFNB2) and autosomal dominant hearing loss (DFNA11) (9). To date, 759 *MYO7A* variants classified as likely pathogenic or pathogenic have been reported in humans (https://www.ncbi.nlm.nih.gov/clinvar; accessed 21 Dec 2023), most of which cause Usher syndrome type 1B. DFNB2 and DFNA11 manifest hearing loss and vestibular dysfunction, but not usually retinal pathology. Patients with Usher syndrome type 1B usually show congenital deafness, vestibular dysfunction, and prepubertal onset RP, which leads to blindness. Although rare, a *MYO7A* variant also correlated with Usher syndrome type 2, which presents less severe features than type 1B (10). CME is not a rare phenotype in *MYO7A*-associated USH, and it is often significantly correlated with alterations in photoreceptor segments (11). In the present study, we report an unusual *MYO7A* mutation-associated phenotype in two patients from one Chinese family, who both present fluctuating CME and rod-cone dysfunction, without other RP-related symptoms or hearing impairment.

## Case description

**Figure 1:**
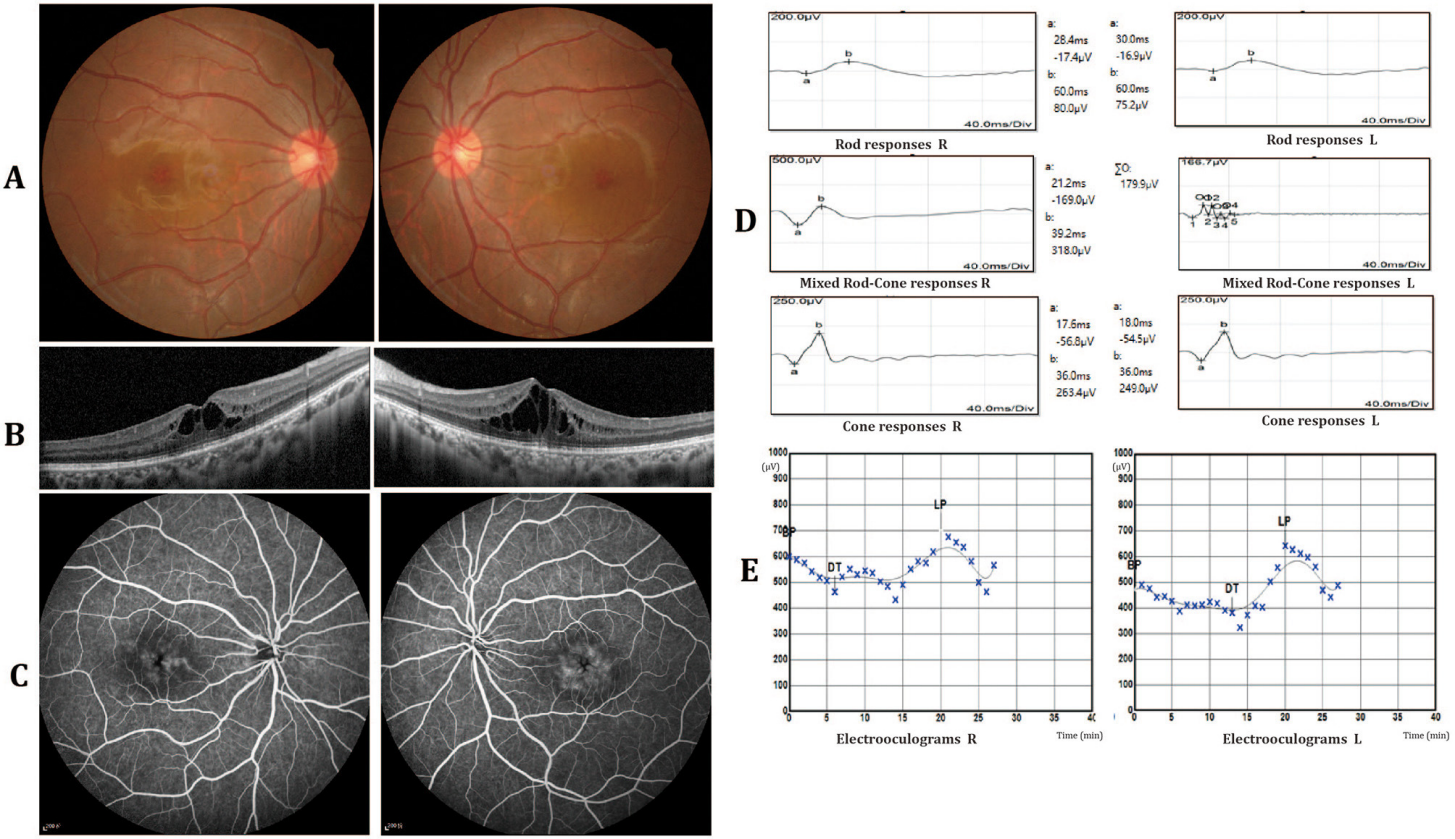
Clinical characteristics of patient II1. **(A)** Fundus photography revealed no abnormalities, with only an absence of the foveal reflex. **(B)** Optical coherence tomography (OCT) showed macular cystoid changes in both eyes. The cystoid cavities are mainly involved in the outer nuclear layer (ONL). **(C)** Fundus fluorescein angiography (FFA) showing cystoid hyperfluorescence in a petaloid pattern in the foveal area with a honeycomb pattern parafoveally. No leakage or abnormal fluorescence change was present in the periphery of the retina. **(D)** Full-field electroretinography (ffERG) showed rod responses to be notably attenuated and cone responses to be prolonged in both eyes. **(E)** The electrooculography (EOG) arden ratios were mildly reduced in both eyes (right/left 1.5/1.7).

Two 23-year-old twin sisters presented with progressive vision deterioration persisting for over 6 months. Clinical data of the two patients II1 and II2 are summarized in Table 1. The family history was negative for ocular diseases, and both patients reported no history of systemic or other ocular diseases since childhood. No abnormalities were observed in the external and anterior segment. Fundus examination revealed no abnormalities, with only an absence of the foveal reflex (Figure 1A). OCT showed macular cystoid changes in both eyes; large and elongated cavities were mainly in the outer nuclear layer (ONL), while smaller, round cystoid cavities were observed at the level of the inner nuclear layer (INL; Figure 1B). Early arteriovenous phase fundus fluorescein angiography (FFA; 4 min) showing cystoid hyperfluorescence in a petaloid pattern in the foveal area with a honeycomb pattern parafoveally. No leakage or abnormal fluorescence change was present in the periphery of the retina (Figure 1C). FfERG showed notable attenuation of rod responses, while cone responses were prolonged in both eyes corresponding to a rod-cone dystrophy dysfunction pattern (Figure 1D). The Arden ratios of electrooculography were mildly reduced (right/left 1.5/1.7) in both eyes, while mfERG showed that the amplitudes of rings 2–5 of both eyes were mild reduced (Figure 1E). Furthermore, all routine laboratory tests related to vasculitis, including erythrocyte sedimentation rate, C-reactive protein (CRP), blood count, serum creatinine, urinalysis, specific autoantibodies, complement, immunoglobulin, cryoglobulin, and hepatitis B and C serology, were negative. The younger sister (II2) showed similar clinical findings (Table 1 and Supplementary Figure S1).

**Table 1:** Clinical characteristics of patients.

**Patient**	**Age (years)**	**Age of onset (years)**	**BCVA, Snellen**	**Refraction**	**Axial length, mm**	**Anterior segment**	**Fundus**	**ffERG**	**mfERG**	**EOG**	**Visual field**	**OCT**	**Fluorescein angiography**
II1	23	22	R:16/20 L:12/20	0.25DS/0DS	22.53/22.57	Normal	Absence of the foveal reflex	Rod responses were notably attenuated, cone responses were prolonged	Amplitudes of rings 2–5 of both eyes were mild reduced	Mildly reduced Arden ratios (1.5/1.7)	Central visual field defect	Macular cystoid changes	Macular fluorescein leakage
II2	23	22	R:12/40 L:12/40	−1.25DS/−1.25DS	22.49/22.39	Normal	Absence of the foveal reflex	Rod responses were notably attenuated, cone responses were prolonged	Amplitudes of rings 2–5 of both eyes were mild reduced	Mildly reduced Arden ratios (2.1/1.7)	Central visual field defect	Macular cystoid changes	Macular fluorescein leakage

**Figure 2:**
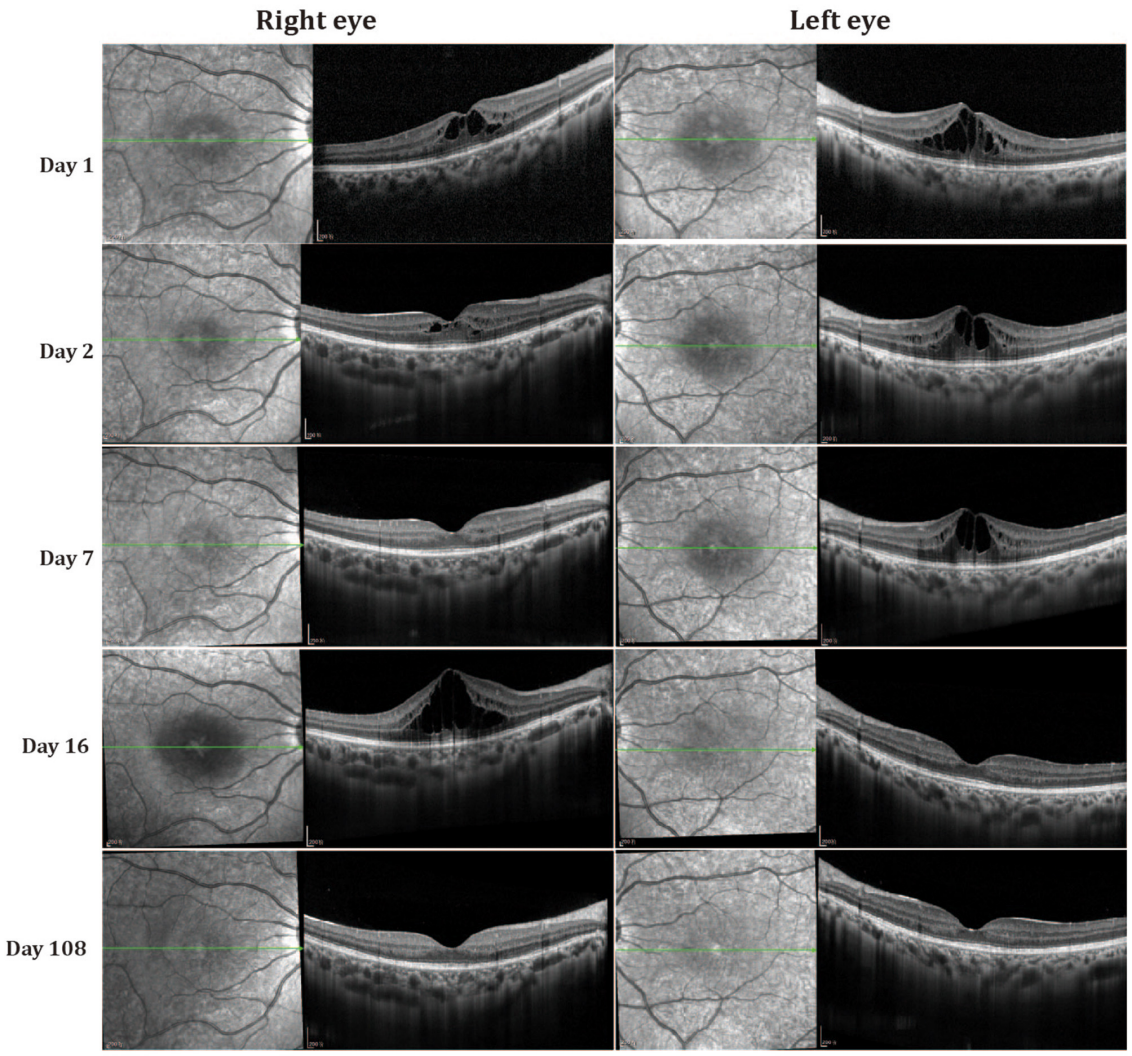
Optical coherence tomography (OCT) changes in patient II1 at different follow-up times.

Follow-up data were available for both patients, with a mean follow-up time of 108 days. Fluctuation of cystoid macular edema (CME) was observed in both eyes without any treatment during this period. The degree of CME was decreased on the second day. The intraretinal cystoid spaces in the fovea were almost absent in the right eye on the third visit (seventh day) but were enlarged in the left eye (Figure 2). However, CME recurred and increased in the right eye on the fourth visit (16th day), but was almost absent from both eyes at 108 days. Final best-corrected visual acuity after 108 days was 10/10 in both eyes, although OCT revealed disorganization of the outer retina at the fovea region. The younger sister (II2) also manifested fluctuation of CME (Supplementary Figures S2, S3).

**Figure 3:**
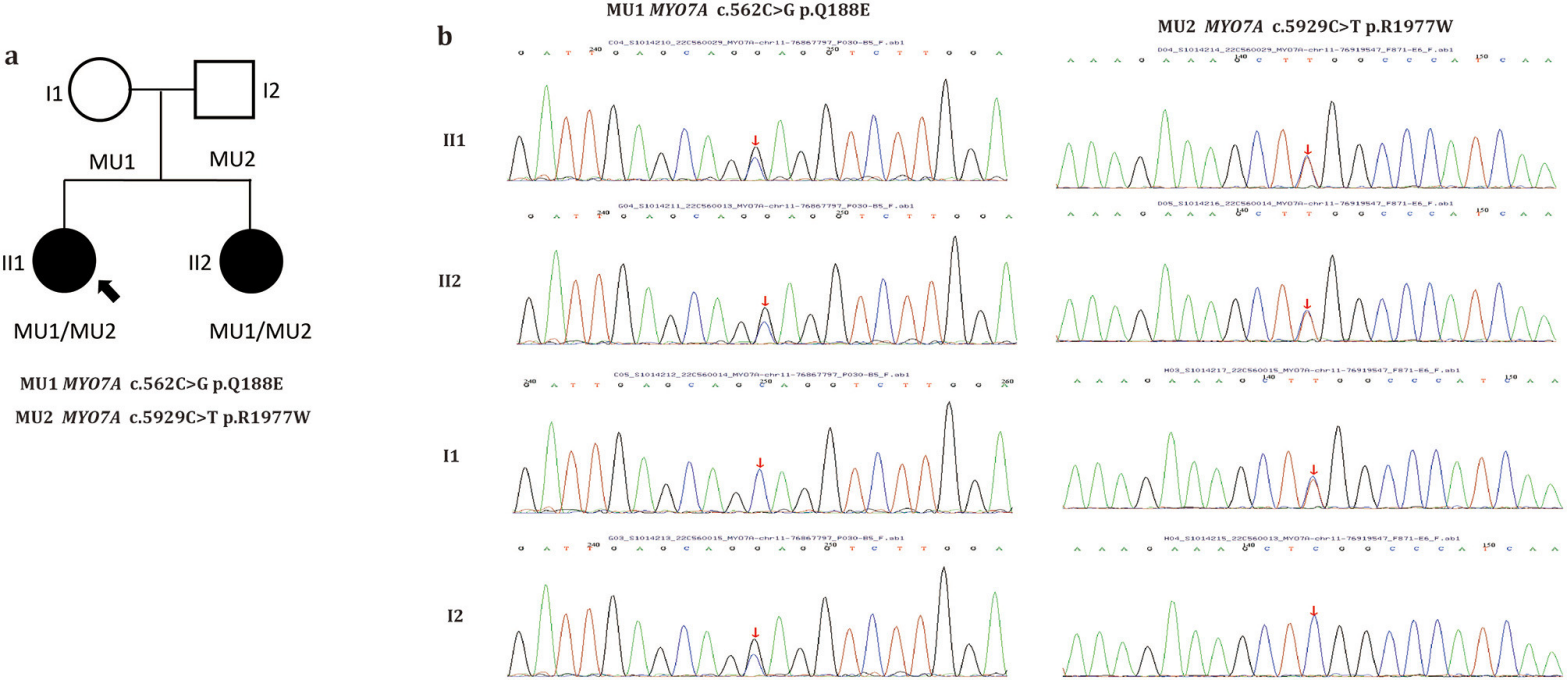
Pedigree of the family **(a)**. Filled symbols indicate patients. Unfilled symbols indicate unaffected family members. Arrow indicates the proband. Squares indicate males and circles females. **(b)** Sanger sequencing of the two *MYO7A* variants identified in this study. Arrows indicate the positions of the mutated nucleotides.

Whole-exome sequencing was performed on the family, and two previously reported mutations, MYO7A c.562C>G p.Q188E (maternally inherited) and MYO7A c.5929C>T p.R1977W (paternally inherited) (12, 13), were found in the two patients (Figures 3a, b). These mutations co-segregated with the phenotype. Of the two mutations, p.R1977W is located in the FERM domain, and p.Q188E is located in the motor domain of the MYO7A protein (Supplementary Figure S4A). Multiple orthologous sequence alignment revealed that MYO7A codon 188 and subsequent sequences encode highly conserved amino acids across different species, indicating that mutation at any of those codons may lead to a deleterious effect (Supplementary Figure S4B). Three-dimensional predictions of the two mutations are shown in Supplementary Figure S4C. Both mutations were extremely rare in the control population. Further analysis using SIFT, MutationTaster, FATHMM, and PolyPhen-2 predicted both mutations to be pathogenic. Details of the weighted allele scores are given in Supplementary Table S1. No additional pathogenic or likely pathogenic variants known to be associated with inherited eye diseases were found in either patient.

## Discussion

We describe a family with an unusual CME phenotype associated with two previously reported mutations in *MYO7A*. The two affected family members were sisters with no ocular history and a negative family history. The appearance of CME in both eyes of both patients was the first and most prominent abnormality, which fluctuated spontaneously during the follow-up period. The retinal pigment epithelium and external limiting membrane bands were intact while the ellipsoid zone was slightly disrupted and disorganized in the central foveal region on OCT images. These cystoid abnormalities corresponded to FFA examinations. Dye diffusion could be detected in the macular area which effectively ruled out a diagnosis of retinoschisis. No vascular leakage was present in the periphery of the retina on FFA images, indicting no uveitis-related CME. Furthermore, all routine laboratory tests related to vasculitis (14), such as EST, CRP, blood count, serum creatinine, urinalysis, specific autoantibodies, complement, immunoglobulin, cryoglobulin, and hepatitis B and C serology, were negative. ERG showed a rod-cone dystrophy dysfunction pattern, and rod function was more impaired than cone function. Furthermore, mfERG showed mild retinal dysfunction and the EOG Arden ratios were mildly reduced. These results, thus indicating rod, cone and retinal pigment epithelial cells were all affected. These changes are different from RP, where the electrophysiological damage is more severe and begins in the peripheral area. Given all this evidence, it is reasonable to speculate that the CME was caused by a breakdown of the barrier function of the RPE. Long-term follow-up is needed to more precisely characterize this family.

The two identified *MYO7A* variants (c.562C>G and c.5929C>T) were considered associated with fluctuation CME for the following reasons. (1) The variants co-segregated with CME in this family. (2) Although the two variants were classified as variants of uncertain significance, they had previously been reported in individuals with hearing loss or Usher syndrome (12, 13), and (3) both mutations were single nucleotide substitutions, c.562C>G leading to p.Gln188Glu and c.5929C>T leading to p.Arg1977Trp. Generation of three-dimensional structures using PyMOL, enabled us to classify the two variants as variants as potentially pathogenic. The challenge in our study was to evaluate the genotype-phenotype correlation of the affected family members. In humans, *MYO7A* is widely localized, such as in cochlea neuroepithelia, vestibular neuroepithelia, retinal photoreceptor cells, and retinal pigment epithelium cells. Mutations in *MYO7A* lead to USH1 in most cases, including a relatively high incidence of CME (15) which was consistent with our affected family members. However, nearly all pathogenic mutations in *MYO7A* are linked to hearing loss but both the proband and their sister presented normal hearing. The molecular and physiological functions of *MYO7A* have been well-characterized; however, clinical heterogeneity and tissue-specific differences of *MYO7A* variants are well-known (7). Patients with an atypical phenotype associated with *MYO7A* are not infrequent, and mutations in *MYO7A* have been associated with diverse clinical phenotypes. Previous studies have indicated that mutations in *MYO7A* might have different effects in the eye but similar effects in the inner ear; however, our patients show that the effects of *MYOYA* variants on the inner ear may also be different. Moreover, *MYO7A* can cause autosomal recessive rod-cone dystrophy in the retina, which may explain why the two patients with fluctuation CME exhibited abnormalities and phenotypic characteristics of rod-cone dysfunction.

There are many reasons why different mutations in the same gene can cause diverse phenotypes. Firstly, *MYO7A* spans ~87 kb of genomic sequence and encodes myosin VIIA, which has a predicted molecular mass of 254 kDa. This protein contains 2,215 amino acids and has three typical domains: the N terminal head or motor (amino acids 1–729); the neck or regulatory domain consisting of five isoleucine glutamine motifs (IQ; IQ1–IQ5: amino acids 745–857); and the tail which begins with a short, coiled-coil domain (amino acids 858–935). These are followed by two large MyTH4-FERM repeat elements, separated by an SH3 domain (amino acids 1,603–1,672) (16). Clinical heterogeneity associated with *MYO7A* variants was recently shown to rely on affected domains. Missense variants in the motor domain present as adult-onset and slowly progressive, leading to a flat configuration, while patients carrying MyTH4 domain variants showed adult-onset, rapid progression, and a down sloping tendency (7). The two compound heterozygous variants found in our pedigree were in different domains of myosin VIIA, one variant was located in the motor domain, and the other in FERM2. Pathogenic variants are most frequently located in motor and MyTH4 domains (17), while variants in FERM2 are rare. Schwander et al. (18) demonstrated that the FERM2 domain of *MYO7A* is associated with the transport function of retinal pigment epithelium cells. They also found that a *MYO7A* point mutation can differentially affect gene expression in the inner ear and retina. These findings might explain why the proband and their sister presented as fluctuating CME, but without hearing impairment. Also consistent with our findings is that compound heterozygous mutations in different domains may cause a mild phenotype (19–21). In addition, a growing number of modifiers have been identified that can affect the phenotype of *MYO7A* variants. Street et al. (22) described a single nucleotide polymorphism as a modifier that contributes to the severe hearing loss phenotype by reducing expression of the wild-type *MYO7A* allele. Morgan et al. (23) showed that PDZD7, which was originally identified as a modifier gene for Usher syndrome type 2, also interacted with *MYO7A*. It is also possible that modifiers may affect the phenotype of our patients.

CME was the first clinically visible abnormality in the two patients, indicating that CME is the direct result of the primary genetic defect. Large and elongated cavities are mainly present in the outer nuclear layer, indicating a primary pathological process affecting retinal photoreceptor cells and retinal pigment epithelium cells. This is supported by ffERG and EOG results, and can be explained by *MYO7A* mutations. As the disease progressed, Müller cell function was disrupted and cystoid fluid accumulation appeared in the inner nuclear layer. It is possible that cell function was not completely destroyed in the early stage leading to CME presenting in a fluctuating form.

This study has some limitations. First, it had a small sample size with only one Chinese family enrolled. Second, we did not explore the effects of the identified variants by molecular investigations. Our future studies will further investigate genotype-phenotype associations of *MYO7A* variants.

In conclusion, we used whole-exome sequencing to identify two compound heterozygous variants in *MYO7A* (both previously reported) that are likely to be associated with fluctuating CME. Our findings expand the phenotypic spectrum of *MYO7A* variants and may enhance understanding of genotype-phenotype associations for compound heterozygous *MYO7A* variants.

## Data Availability

The original contributions presented in the study are included in the article/Supplementary material, further inquiries can be directed to the corresponding authors.

## Supplementary material

Supplementary Figure 1 Fundus photography (A and B), fundus fluorescein angiography (FFA, C and D) and optical coherence tomography (OCT, E and F) findings of patient II2. FFA showing cystoid hyperfluorescence in a petaloid pattern in the foveal area with a honeycomb pattern parafoveally. OCT showed macular cystoid changes in both eyes, and cystoid cavities mainly in the outer nuclear layer (ONL).

Supplementary Figure 2 Optical coherence tomography (OCT) changes in patient II1 at different follow-up times.

Supplementary Figure 3 Fundus fluorescein angiography (FFA) changes in patient II2 at different follow-up times.

Supplementary Figure 4 Location of the variants in the MYO7A protein domains (A). Alignment of human *MYO7A* protein sequence (B) with orthologs. Three-dimensional prediction of the two mutations (variants and wild-type, C). II2 at different follow-up times.
