# Supplementary figures and images for: Case Report: A family of fluctuating cystoid macular edema caused by MYO7A gene mutations
Source: Front Med (Lausanne). 2025 Aug 7;12:1582930. doi: 10.3389/fmed.2025.1582930 (PMC12367652; doi:10.3389/fmed.2025.1582930)

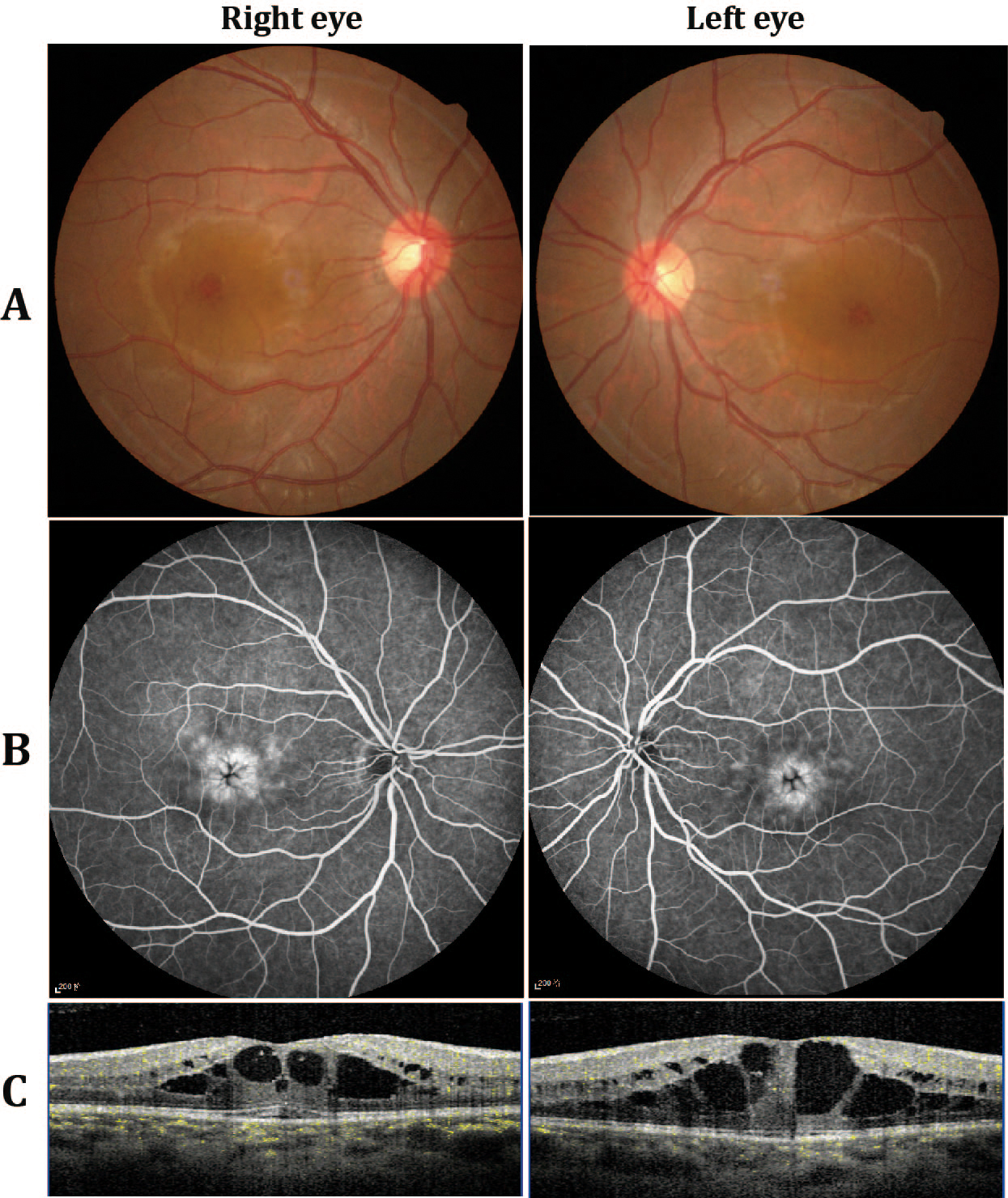

Supplement: Supplementary Figure 1 — Fundus photography (A and B), fundus fluorescein angiography (FFA, C and D) and optical coherence tomography (OCT, E and F) findings of patient II2. FFA showing cystoid hyperfluorescence in a petaloid pattern in the foveal area with a honeycomb pattern parafoveally. OCT showed macular cystoid changes in both eyes, and cystoid cavities mainly in the outer nuclear layer (ONL). [file Image_1.jpeg]

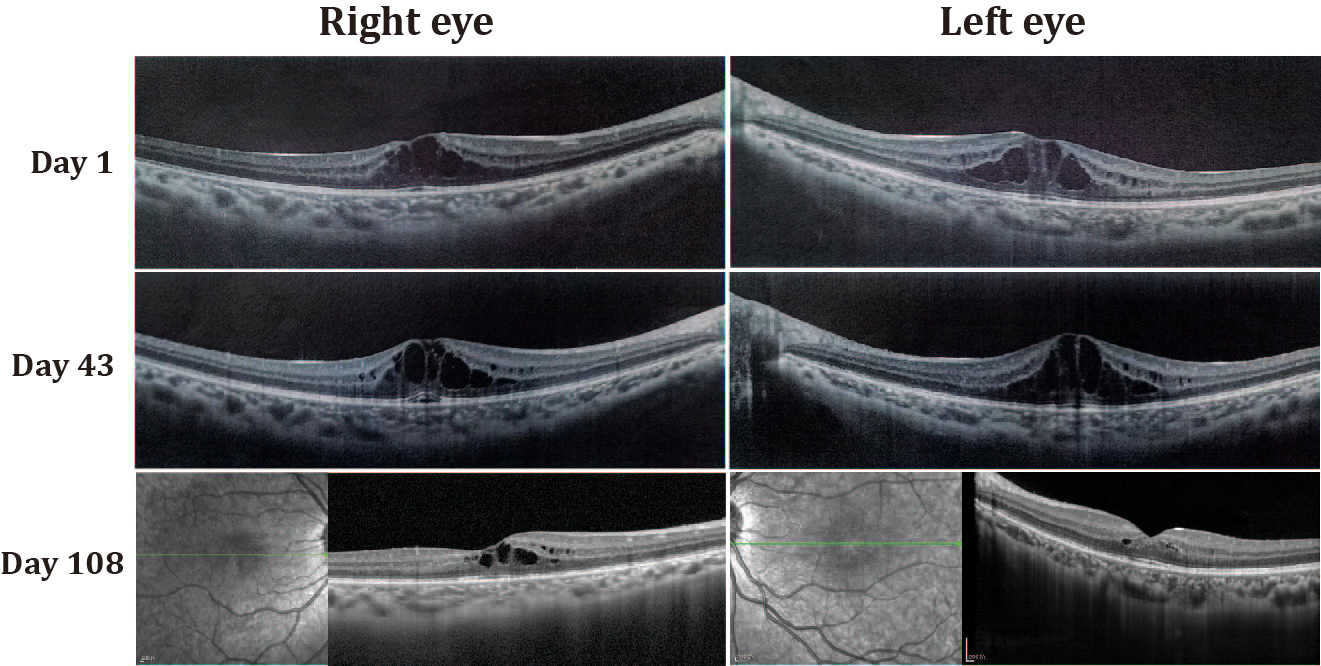

Supplement: Supplementary Figure 2 — Optical coherence tomography (OCT) changes in patient II1 at different follow-up times. [file Image_2.jpeg]

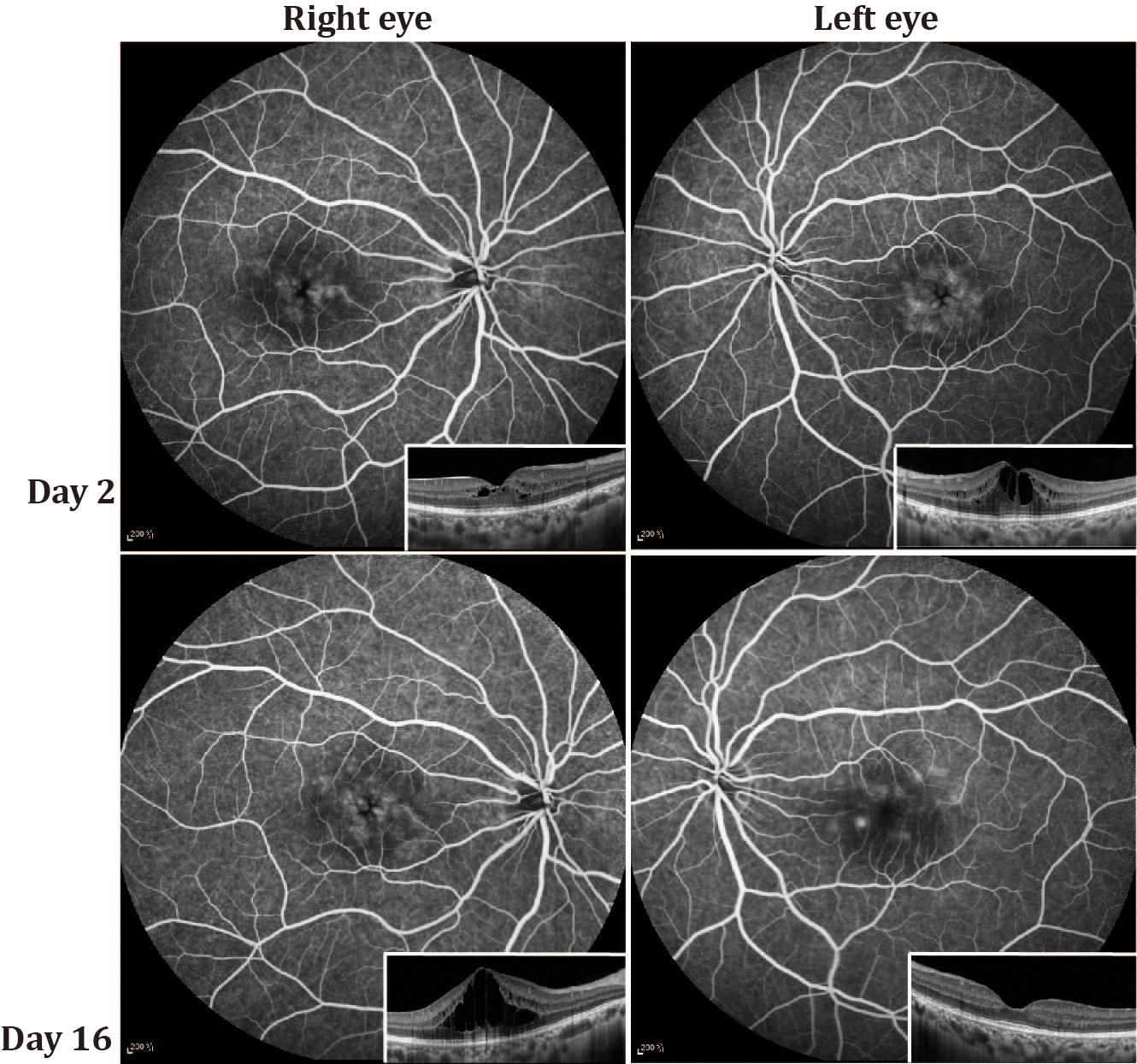

Supplement: Supplementary Figure 3 — Fundus fluorescein angiography (FFA) changes in patient II2 at different follow-up times. [file Image_3.jpeg]

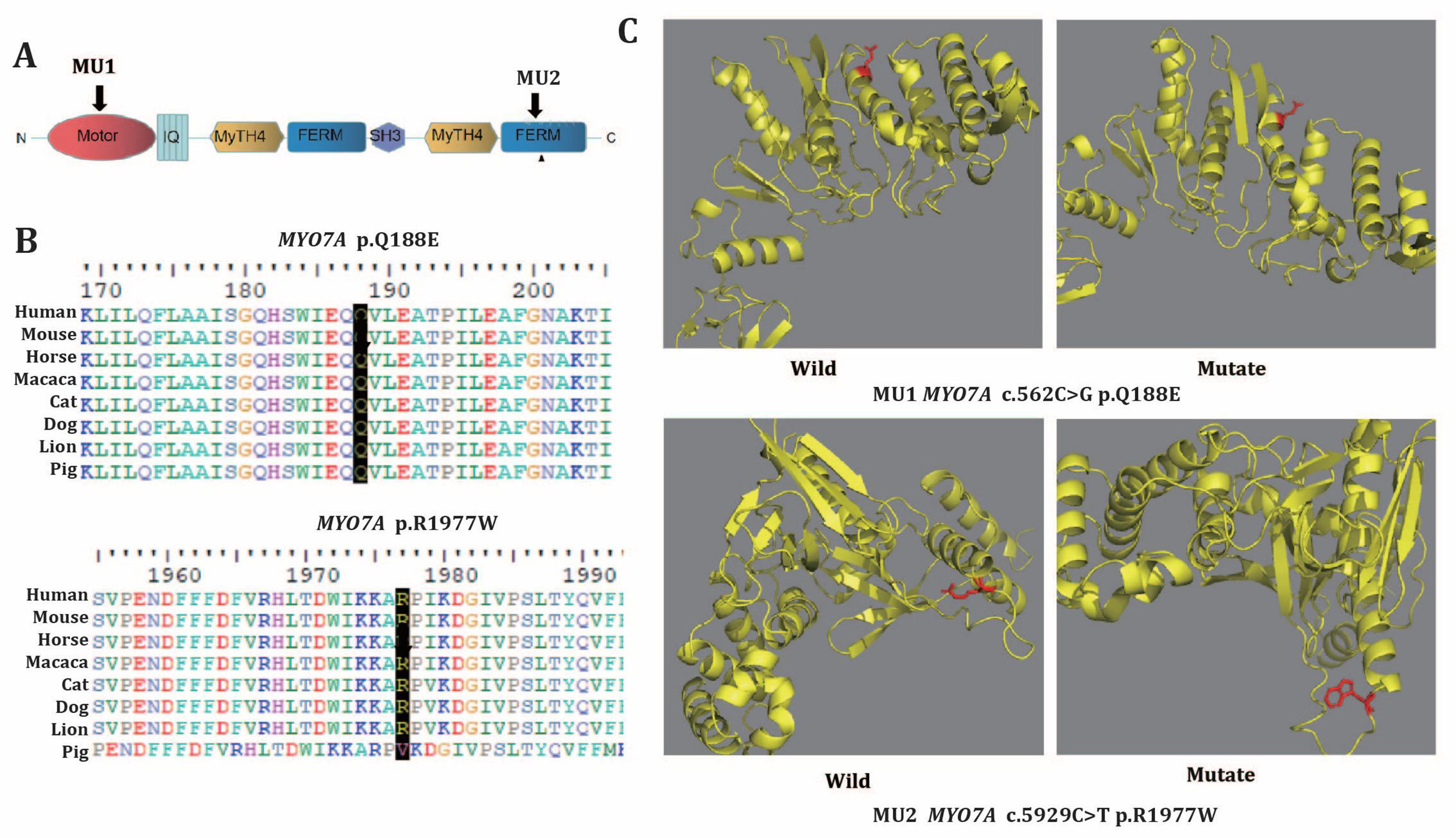

Supplement: Supplementary Figure 4 — Location of the variants in the MYO7A protein domains (A). Alignment of human MYO7A protein sequence (B) with orthologs. Three-dimensional prediction of the two mutations (variants and wild-type, C). II2 at different follow-up times. [file Image_4.jpeg]
