# Supplementary material for: Case Report: A family of fluctuating cystoid macular edema caused by MYO7A gene mutations
Source: Front Med (Lausanne). 2025 Aug 7;12:1582930. doi: 10.3389/fmed.2025.1582930 (PMC12367652; doi:10.3389/fmed.2025.1582930)
Supplement: Supplementary file 5 [file Table_1.docx]

Table S1. Bioinformatics analysis results of the two variants in *MYO7A* gene.

| Amino acid Change | Nucleotide Change | SIFT | MutationTaster | FATHMM | PolyPhen_2_ | 1000g2015 | ExAC |
| --- | --- | --- | --- | --- | --- | --- | --- |
| Arg1977Trp | 5929C>T | Damaging | Disease_causing | Damaging | Probably_damaging | - | 0.00001843 |
| Gln188Glu | 562C>G | Damaging | Disease_causing | Damaging | Probably_damaging | 0.000199681 | 0.0000497 |
